# Myocarditis in SARS-CoV-2 infection vs. COVID-19 vaccination: A systematic review and meta-analysis

**DOI:** 10.3389/fcvm.2022.951314

**Published:** 2022-08-29

**Authors:** Navya Voleti, Surya Prakash Reddy, Paddy Ssentongo

**Affiliations:** ^1^Department of Medicine, Penn State Health Medical Center, Hershey, PA, United States; ^2^Department of Medicine, Osmania Medical College, Hyderabad, India; ^3^Department of Public Health Sciences, Penn State College of Medicine, Hershey, PA, United States

**Keywords:** myocarditis, SARS-CoV-2, COVID-19 vaccines, global health, cardiovascular medicine

## Abstract

**Background:**

This study aimed to compare the incidence of myocarditis in COVID-19 vaccines and in severe acute respiratory syndrome-coronavirus 2 (SARS-CoV-2) infection groups.

**Methods:**

Electronic databases (MEDLINE, Scopus, Cochrane Central Register of Controlled Trials, Cochrane Database of Systematic Reviews, and the WHO Global Literature on Coronavirus Disease) and trial registries were searched up to May 2022, for randomized controlled trials and observational cohort studies reporting the risk of myocarditis associated with the COVID-19 vaccines and the risk associated with SARS-CoV-2 infection. We estimated the effect of COVID-19 infection and vaccines on rates of myocarditis by random-effects meta-analyses using the generic inverse variance method. Meta-regression analyses were conducted to assess the effect of sex and age on the incidence of myocarditis.

**Results:**

We identified 22 eligible studies consisting of 55.5 million vaccinated cohorts and 2.5 million in the infection cohort. The median age was 49 years (interquartile range (IQR): 38–56), and 49% (IQR: 43 to 52%) were men. Of patients diagnosed with myocarditis (in both vaccination and COVID-19 cohort) 1.07% were hospitalized and 0.015% died. The relative risk (RR) for myocarditis was more than seven times higher in the infection group than in the vaccination group [RR: 15 (95% CI: 11.09–19.81, infection group] and RR: 2 (95% CI: 1.44-2.65, vaccine group). Of patients who developed myocarditis after receiving the vaccine or having the infection, 61% (IQR: 39–87%) were men. Meta-regression analysis indicated that men and younger populations had a higher risk of myocarditis. A slow decline in the rates of myocarditis was observed as a function of time from vaccination. The risk of bias was low.

**Conclusion:**

In this systematic review and meta-analysis, we found that the risk of myocarditis is more than seven fold higher in persons who were infected with the SARS-CoV-2 than in those who received the vaccine. These findings support the continued use of mRNA COVID-19 vaccines among all eligible persons per CDC and WHO recommendations.

## Introduction

The severe acute respiratory syndrome coronavirus 2 (SARS-CoV-2) is a strain that causes COVID-19. As of 15 May 2022, more than 520 million COVID-19 cases and 6.3 million deaths were reported globally (1). To combat the high burden of COVID-19, vaccines were introduced to reduce the risk of severe illness and deaths (2). Although the vaccines have proven to reduce severe COVID-19, cardiac complications, particularly myocarditis and pericarditis, have been associated with mRNA COVID-19 vaccination (3–5). On the other hand, myocarditis is also one of the complications of SARS-CoV-2 infection (3, 6). However, the relative risk of myocarditis due to vaccines and infections is not well characterized. In severe form, myocarditis can result in chronic heart failure or death, which are important safety concerns. Given the high rate of vaccine hesitancy due to the fear of vaccine-induced serious adverse events such as cardiac complications, it is critical to characterize the relative risk of vaccine- and infection-induced myocarditis in the general population and determine the effect of sex and age on the risk. In this systematic review and meta-analysis, we estimate the incidence of myocarditis due to SARS-CoV-2 infection vs. COVID-19 vaccination.

## Methods

Results were reported following Preferred Reporting Items for Systematic Reviews and Meta-analyses (PRISMA) 2020 (7).

### Data sources and searches

Databases such as the MEDLINE, Scopus, Cochrane Central Register of Controlled Trials, Cochrane Database of Systematic Reviews, the WHO Global Literature on Coronavirus Disease, and CoronaCentral were searched from December 2019 to May 2022, without language restriction. Clinical trial registries and conference proceedings were also searched. The following Medical Subject Headings and keyword search terms were used; [“myocarditis” OR cardiac complications ] AND [“SARS-CoV-2” OR “Covid-19” OR “severe acute respiratory syndrome coronavirus-2” OR “coronavirus disease 2019”] AND “vaccines”.

### Study selection

Studies were selected according to Participant (P) Intervention/Exposure (I/E) Comparator [C], Outcome (O) Study type (S) [PI(E)COS] criteria (8):

**Participants:** Persons of all ages and sex included in studies that reported cardiac complications in either COVID-19 vaccines or due to COVID-19 infection group.
**Intervention/Exposure**: 1) COVID-19 vaccines and 2) SARS-CoV-2 infection.
**Comparison:** 1) Non-vaccinated group and 2) Individuals without infection.
**Outcome of interest**: Myocarditis.
**Study type:** Randomized clinical trials (RCT) and observational studies.

Pairs of independent investigators (NV and SPR) screened the titles and abstracts of all citations. Studies included by either reviewer were retrieved for full-text screening. Independent investigators (NV and SPR) screened the full-text version of eligible studies. Disagreements in the included papers were resolved by discussion and if necessary, a third investigator (PS) was consulted.

### Data extraction and quality assessment

A standardized data extraction form was developed, and two investigators (NV and SPR) worked independently to extract study details. The following information was extracted: year of study publication, country, study design, study-level descriptive statistics (mean (SD)/median (IQR) age in years, and proportion (%) of women and men), median/mean follow-up, the type of vaccine, numbers of myocarditis cases following infection and vaccines, the relative risk of myocarditis. The risk of bias was evaluated at the outcome level using the Cochrane Collaboration tool for RCTs and Newcastle-Ottawa Scale for observational studies (9, 10). Observational studies with fewer than 5 stars were considered low quality (high risk of bias); 5 to 7 stars, moderate quality (some concerns); more than 7 stars, high quality (low risk of bias). RCTs' risk of bias was categorized as low, or some concerns.

### Data synthesis and analysis

Statistical analyses were performed with R software version 3.6.2 (R Project for Statistical Computing). The *Meta* and *Metafor* R packages were used to conduct formal meta-analyses and create forest plots. Descriptive statistics were used to summarize study-level demographics.

The primary outcome was the myocarditis risk due to the vaccines and SARS-CoV-2 infection. Effect sizes were log-transformed to normalize the distributions. Standard errors (SEs) were calculated *via* the following equations (11): Lower = log (lower 95% CI) and upper = log (upper 95% CI), and SE = (upper-lower)/3.92. To determine the effect of sex and age on the rates of myocarditis, we conducted a univariate meta-regression analysis with the mean (or median) age of each study and the proportion of men in the study as regressors.

The pooled RR estimates for myocarditis risk from each study were weighted by the inverse of their variances (inter-study plus intra-study variances). The DerSimonian and Laird's (DL) random-effects method was used to estimate the pooled inter-study variance (heterogeneity) (12). Heterogeneity between studies was evaluated with the *I*^2^ indicator expressed as percent low (25%), moderate (50%), and high (75%) (13).

Publication bias was quantitatively evaluated with Egger's linear regression and Begg's rank test (14, 15) and qualitatively with funnel plots. Trim and fill analyses using Duval and Tweedie's non-parametric method were used to adjust for the publication bias (16). Two-sided *p* < 0.05 was deemed statistically significant.

## Results

### Identified studies

**Figure 1:**
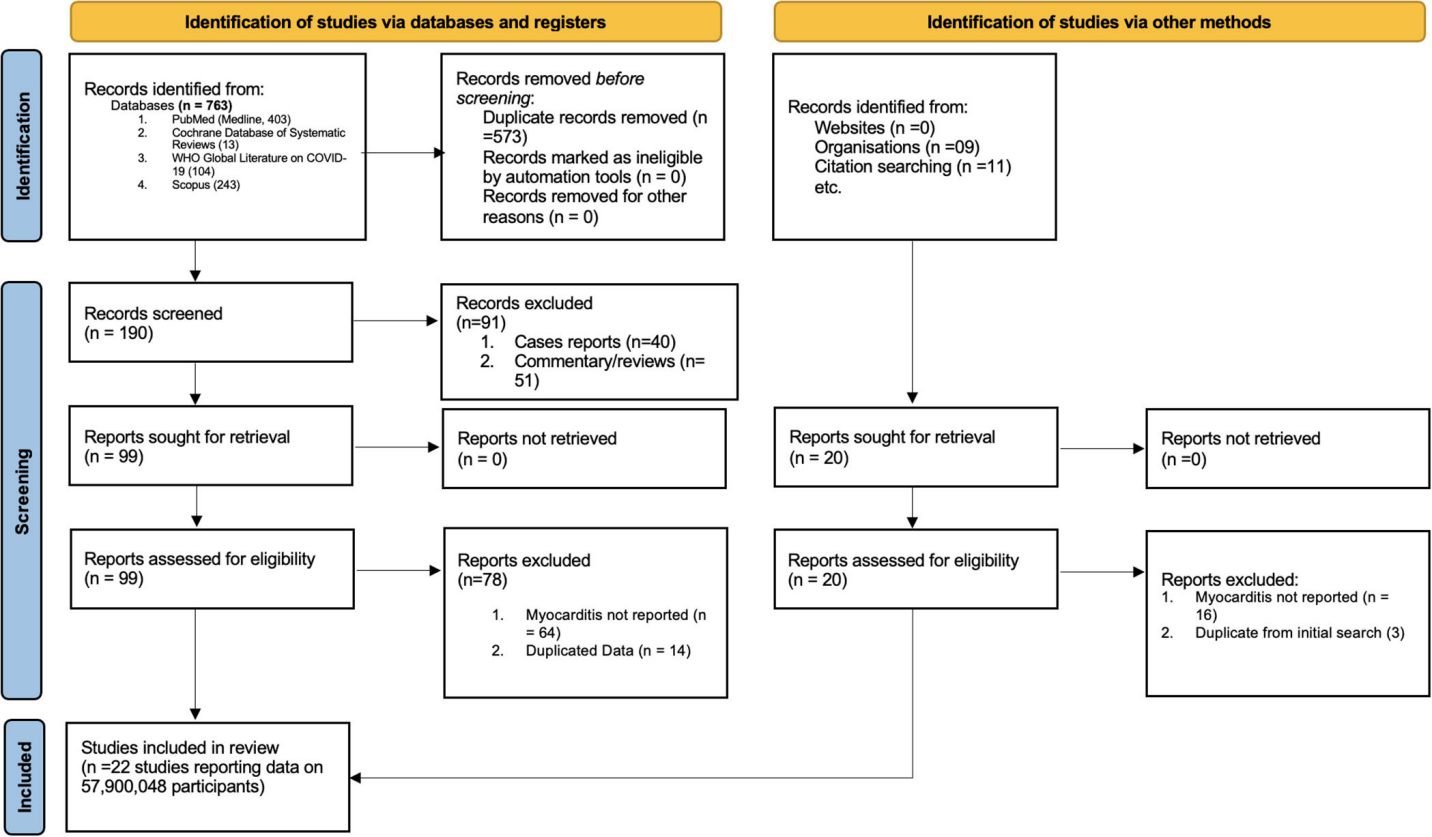
PRISMA flow diagram for study selection.

The study selection process is shown in Figure 1. A total of 763 studies were screened. The exclusion process yielded 22 studies conducted in eight countries and three WHO regions. The baseline characteristics of the studies included in the meta-analysis are presented in Table 1. Included studies consisted of 58 million persons, with 55.5 million in the vaccination cohort and 2.5 million in the infection cohort (Table 1). Overall, median age was 49 years (interquartile range (IQR): 38–56), and 49% (IQR: 43–52%) were men. Then, 10 studies were assessed for myocarditis rates from infection and 12 studies from COVID-19 vaccines. Of the vaccine studies, eight assessed mRNA vaccines (Pfizer and Moderna), one study Novavax, one study adenovirus vectors (AstraZeneca), and one study combined mRNA and J and J vaccine. Of patients diagnosed with myocarditis (in both vaccination and COVID-19 cohort) 1.07% were hospitalized and 0.015% died. Of patients who developed myocarditis after receiving the vaccine or having the infection, 61% (IQR: 39–87%) were men. Of patients diagnosed with myocarditis (in both vaccination and COVID-19 cohort) 1.07% were hospitalized and 0.015% died. The median follow-up time from infection or vaccine to myocarditis was 28 days (IQR: 28–30 days). The median study quality score among the observational studies was 8 (range: 7–9) and was deemed as having a low risk of bias. Similarly, RCTs also had a low risk of bias.

**Table 1:** Study level characteristics.

**References**	**Publication year**	**Country**	**Cohort**	**Risk of bias**	**Age, y (mean)**	**Male (% total)**	**Male (% myocar- ditis)**	**Vaccine type**	**Sample size**	**Follow-up (day)**	**Myocarditis (n)**	**Myocarditis diagnostics**	**Male (% myocarditis)**	**Hospitalization (n)**	**Deaths (n)**
Mevorach et al. (17)	2021	Israel	Vaccine	Low		49	91	Pfizer	5000000	30	136	Clinical (Brighton classification)	91	114	1
Witberg et al. (4)	2021	Israel	Vaccine	Low	44	49	94	Pfizer	2500000	42	54	Clinical (CDC definition)	94	1	1
Barda et al. (18)	2021	Israel	Vaccine	Low	38	52	91	Pfizer	884828	42	21	Clinical (CHS billing criteria)	91		
Walter et al. (19)	2022	US	Vaccine	Low	8	52	0	Pfizer	1518	7	0	N/A	0	0	0
El Sahly et al. (20)	2021	US	Vaccine	Low	51	53	0	Moderna	14287	14	0	N/A	0	0	0
Heath et al. (21)	2021	UK	Vaccine	Low	56	52		Novavax	7569	28	0	Clinical		1	0
Ali et al. (22)	2021	US	Vaccine	Low	14	52	0	Moderna	2489	83	1	N/A	0	0	0
Dunkle et al. (23)	2022	US/Mexico	Vaccine	Low	47	52	0	Novavax	19714	90	0	N/A	0	0	0
Diaz et al. (24)	2022	US	Vaccine	Low	57	41	75	Pfizer/Moderna/J&J	2000287		20	Abnormal troponin or CMR^*^ evidence	75	19	0
Simone et al. et al. (25)	2021	US	Vaccine	Low	49	46	100	Pfizer/Moderna	2392924	10	15	Clinical/Diagnosis code	100	0	0
Husby et al. (26)	2022	Denmark	Vaccine	Low			73	Pfizer	3482295	28	48	Clinical diagnosis + troponin elevation + hospitalization for > 24hours	73	28	1
Husby et al. (26)	2022	Denmark	Vaccine	Low				Moderna	498814	28	21	Clinical diagnosis + troponin elevation + hospitalization for > 24hours		8	0
Patone et al. (3)	2022	UK	Vaccine	Low	55	35	100	AstraZeneca	20615911	28	226	Hospital admission codes	100		
Patone et al. (3)	2022	UK	Vaccine	Low	56	33		Pfizer	16993389	28	158	Hospital admission codes			
Patone et al. (3)	2022	UK	Vaccine	Low	40	26		Moderna	1006191	28	9	Hospital admission codes			
Huang et al. (27)	2020	China	SARS-CoV-2 Infection	Some concerns	38	38	38		26	50	15	CMR	38		
Lagana et al. (28)	2021	Italy	SARS-CoV-2 Infection	Some concerns	71	48	42		1169		12	Clinical = 1 criteria from ESC guidelines	42	1169	3
Boehmer et al. (6)	2021	US	SARS-CoV-2 Infection	Low	54	59	42		1452773	30	5,069	N/A	42		
Murk et al. (29)	2020	US	SARS-CoV-2 Infection	Low	65	43			70288	30		Diagnosis code			
Kunal et al. (30)	2020	India	SARS-CoV-2 Infection	Low	51	65	50		108	7	3	Definite diagnosis with biopsy, probably diagnosis with clinical characteristics with either troponin elevation, myocardial injury, or ECG changes suggestive of injury	50		1
Deng et al. (31)	2020	China	SARS-CoV-2 Infection	Low	65	51	71		112	66	14	AHA guideline: triple elevation in troponin with either ECG changes or echocardiographic changes	71	112	13
Daniels et al. (32)	2021	US	SARS-CoV-2 Infection	Low		67	73		2810	77	37	CMR	73		0
Martinez et al. (33)	2021	US	SARS-CoV-2 Infection	Some concerns	25	99			789	19	3	CMR		0	0
Buckley et al. (34)	2021	US	SARS-CoV-2 Infection	Low	48	43	44		718365		35,820	N/A	44		
Barda et al. (18)	2021	Israel	SARS-CoV-2 Infection	Low	34	46			233392		93,812	N/A: diagnosis code			

* CMR = Cardiac Magnetic Resonance Imaging; J&J, Johnson and Johnson.

### Risk of myocarditis due to SARS-CoV-2 infection vs. COVID-19 vaccination

**Figure 2:**
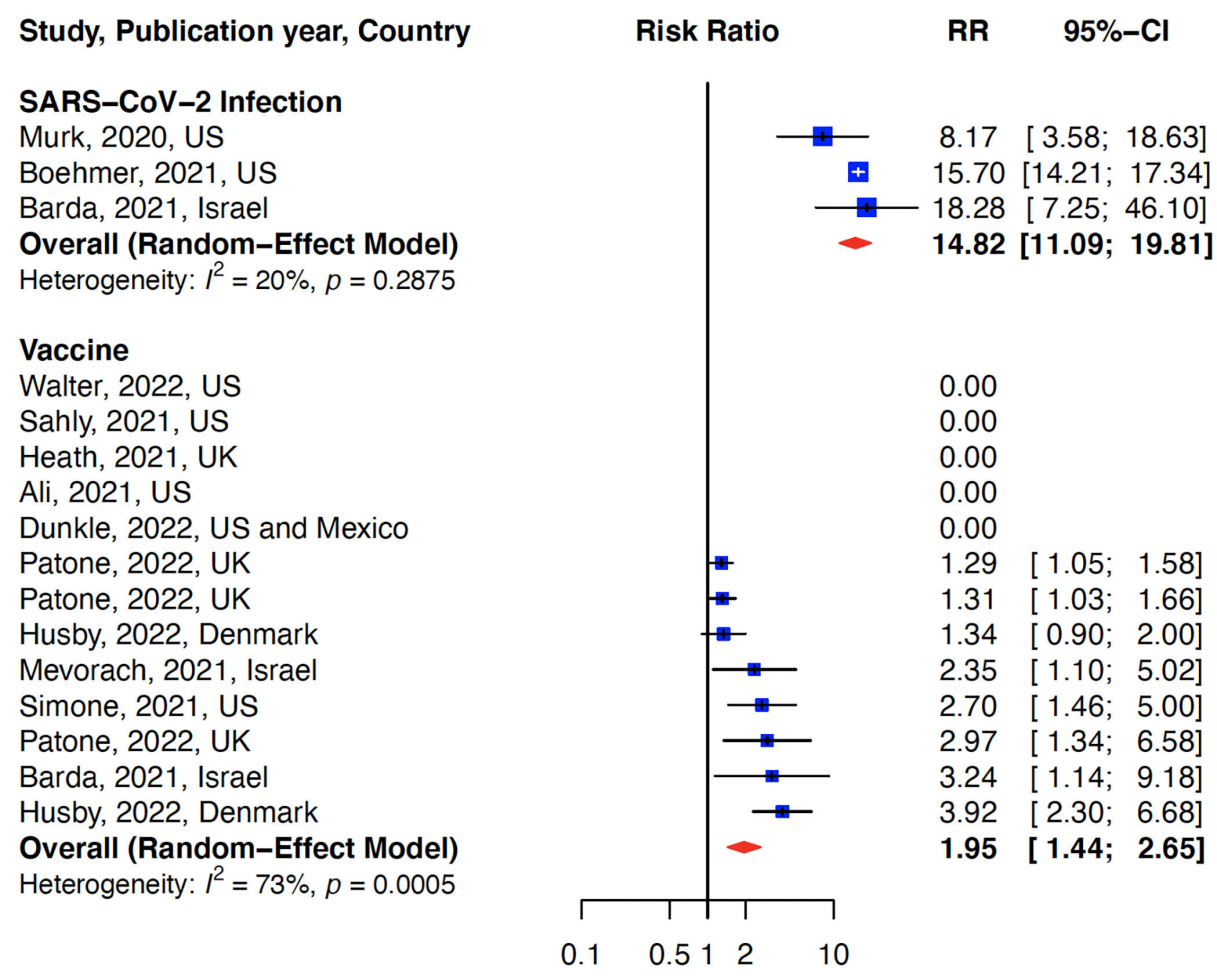
Association of myocarditis after COVID-19 vaccination versus SARS-CoV-2 infection. The risk of myocarditis from infection was more than 7-fold higher than vaccination.

**Figure 3:**
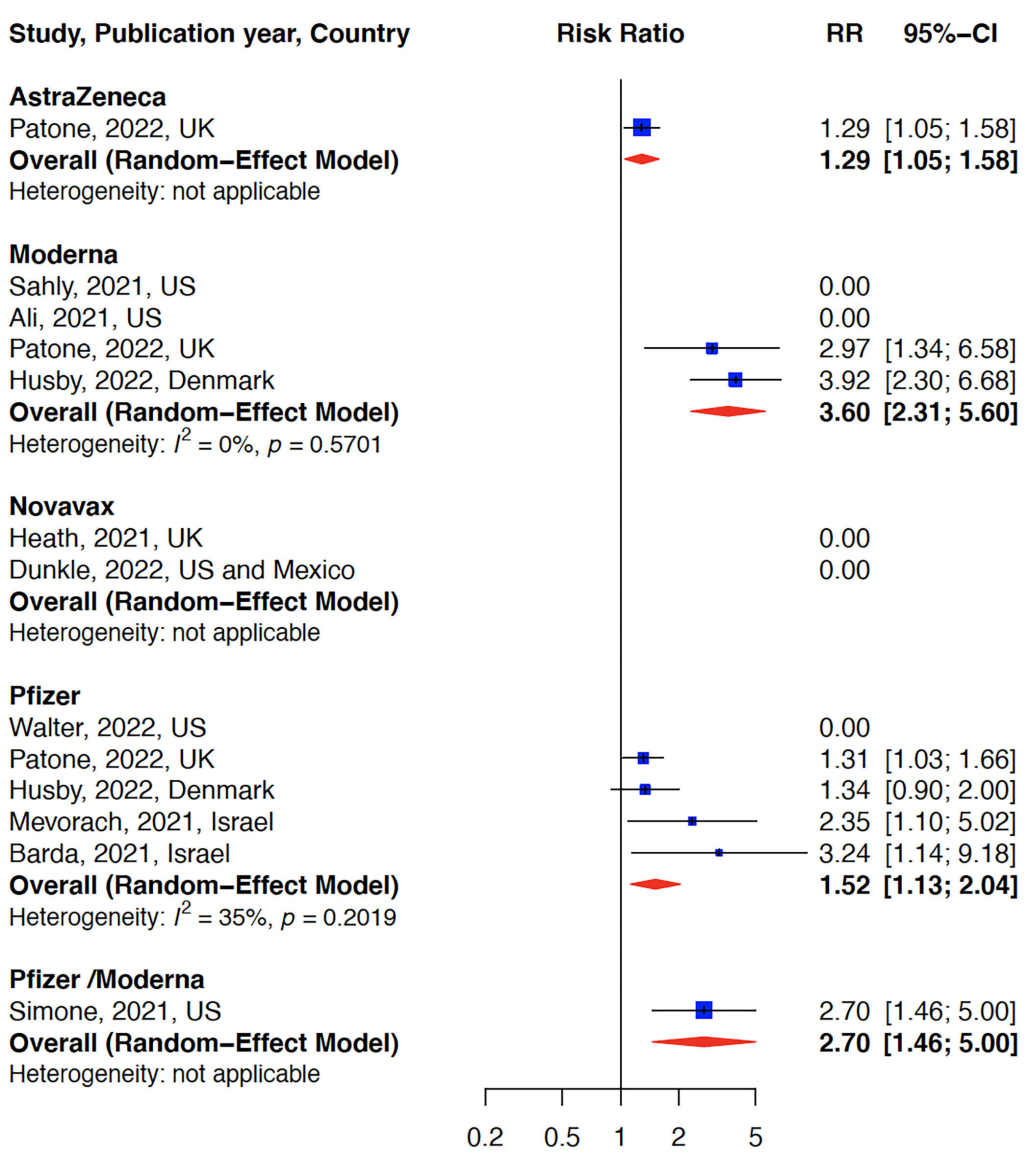
Myocarditis after COVID-19 vaccination stratified by vaccine type. The risk of myocarditis was highest in the Moderna vaccine group.

**Figure 4:**
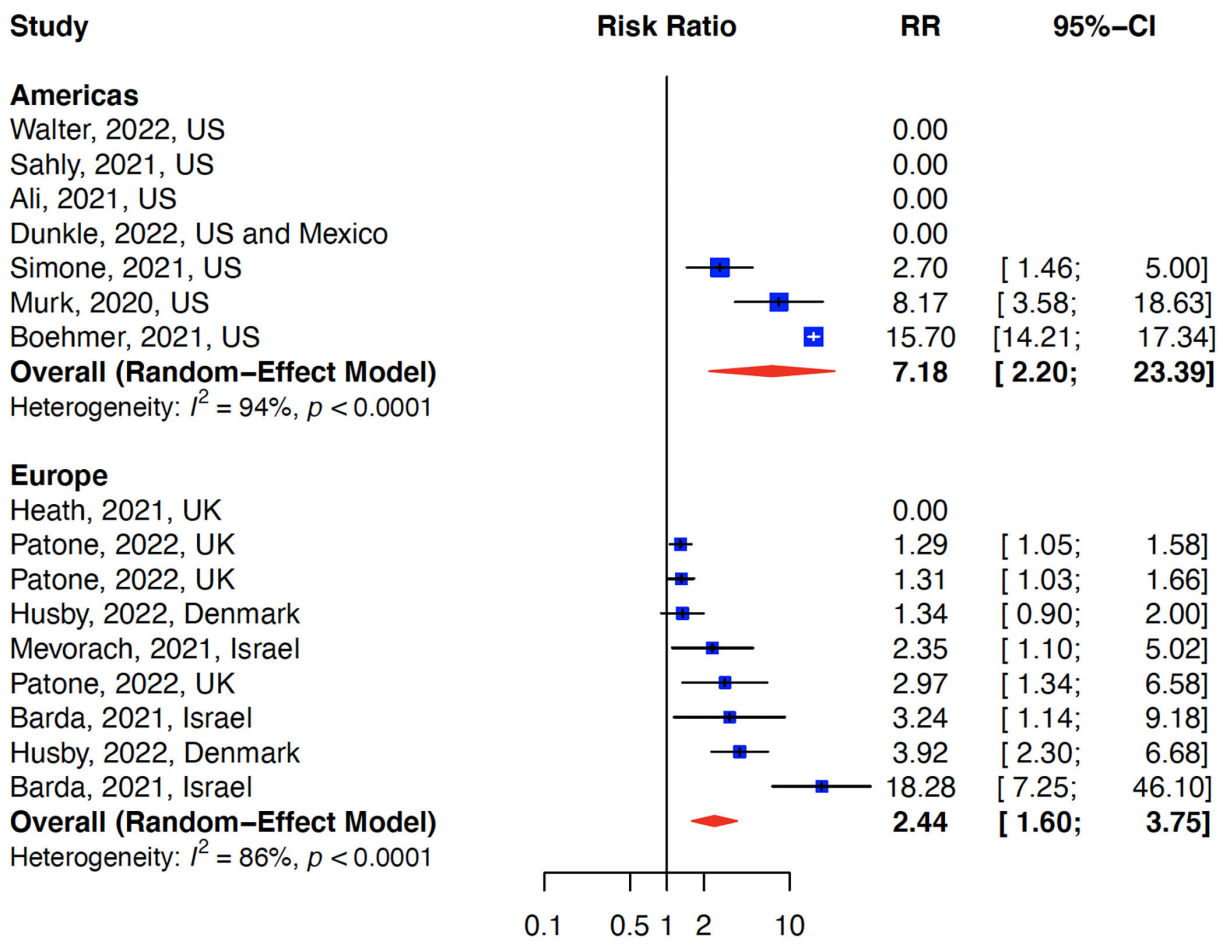
Myocarditis after COVID-19 vaccination/Infection stratified by WHO regions. The risk of myocarditis was higher in the Americas (US and Mexico) compared to Europe.

The relative risk (RR) for myocarditis was more than seven times higher in the infection group than vaccination group (RR: 15 (95% CI: 11.09–19.81, infection group) and RR: 2 (95% CI: 1.44–2.65), vaccine group, Figure 2). Higher rates of myocarditis were observed in those who received Moderna vaccines followed by Pfizer vaccines and the lowest in other vaccines groups (Figure 3). Additionally, higher rates of myocarditis were observed in studies conducted in the Americas (the United States and Mexico) compared to other WHO regions (Figure 4).

### Meta-regression, publication bias, and study heterogeneity

To assess the effect of sex, age, types of vaccines (mRNA vs. non-mRNA vaccines), WHO regions, and follow-up time on myocarditis, we carried out a univariate meta-regression. The analysis was stratified by vaccine and infection risk rates separately. In the studies that examined vaccine risk ratios, younger age was associated with the increasing risk of myocarditis. Although male sex, mRNA vaccines, and studies conducted in the Americas were associated with an increased risk of myocarditis, the association did not reach statistical significance (Table 2). When vaccines and infection studies were combined, male sex and the Americas WHO region were associated with an increased risk of myocarditis, but age and follow-up time were not.

**Table 2:** Results of meta-regression analyses.

**Cohorts**	**Covariate**	**N studies**	**RR**	***p-***	**R^2^**
			**(95% CI)**	**value**	**(%)**
Only vaccine studies	% male (per 5%-point increase in prop male)	6	1.15 (0.96 to 1.37)	0.13	40
	Age (per 5-year increase)	5	0.74 (0.61 to 0.89)	**0.002**	100
	Follow-up (per 7-d increase from vaccination)	8	0.97 (0.71 to 1.13)	0.87	0.0
	mRNA vaccines vs. other	8	1.70 (0.67 to 4.31)	0.26	0.0
	Americas	7	1.45 (0.56 to 3.75)	0.44	6.0
Vaccines and infection studies	% male (per 5%-point increase in prop male)	9	1.38 (1.14 to 1.68)	**0.0009**	89
	Age (per 5-y increase)	8	0.98 (0.88 to 1.10)	0.76	0
	Follow-up (per 7-d increase from vaccination)	10	1.11 (0.45 to 2.74)	0.82	6
	Americas vs. other WHO regions	11	2.94 (1.17 to 7.44)	**0.02**	82

Bold values are statistically significant. R^2^: Coefficient of determination represents the amount of variation in the risk of myocarditis explained by the covariate in the meta-regression model.

Publication bias was assessed only for the vaccine cohort studies. Due to the small number of studies in the infection cohort, we did not assess publication bias for these studies. Visual inspection of a funnel plot of the included studies did indicate a slight asymmetry indicative of mild publication bias. Egger's test for publication bias was significant (p=0.01) but Begg's test was non-significant (*p* = 0.14). Consequently, we conducted Duval and Tweedie's trim and fill test to balance the funnel plot and adjust for potential publication bias (16). The results indicated that if publication bias existed, three additional studies would be needed to eliminate bias, and the overall effect of vaccines on myocarditis would change from 2 (95% CI: 1.44–2.65) to 1.5 (95% CI: 1.11–1.26, Supplementary Figure S1). To identify outlier studies, we further performed influence sensitivity analyses by excluding and replacing one study at a time (Leave-One-Out method) from the meta-analysis and calculated the RR for the remaining studies (35). No substantial change from any of the pooled RR was observed when other studies were removed in turn indicating that no individual study had a considerable influence on the pooled estimate. The plots for the analysis estimates are provided in Supplementary Figure S2.

## Discussion

This is the first systematic review and meta-analysis and the largest study to date of acute myocarditis after SARS-CoV-2 vaccination or infection that estimate the risk ratio of myocarditis due to SARS-CoV-2 infection vs. COVID-19 vaccination. We found that the risk of myocarditis increased by a factor of 2 and 15 after vaccination and infection, respectively. This translates into more than a 7-fold higher risk in the infection group compared to the vaccination group. Among the persons with myocarditis in the vaccinated group, 61% (IQR: 39–87%) were men. Younger populations demonstrated an increased risk of myocarditis after receiving the COVID-19 vaccination. Nevertheless, the risk of hospitalization and death was low. This review is important as there is much hesitancy in the general population of receiving the COVID-19 vaccine given its serious adverse effects.

Our findings are consistent with the recent analysis of EHR data from 40 U.S. healthcare systems which found the incidences of cardiac complications after SARS-CoV-2 infection of nearly 7-fold higher than after mRNA COVID-19 vaccination (36). The risk was higher for both men and women in all age groups. In a Danish population study, vaccination with mRNA-1273 was associated with a significantly increased risk of myocarditis, primarily driven by an increased risk among individuals aged 12–39 years (26). Nevertheless, the absolute rate of myocarditis or myopericarditis after SARS-CoV-2 mRNA vaccination was low, even in younger age groups.

Myocarditis can be self-limiting, but some cases can advance to life-threatening conditions with associated heart failure, arrhythmias, or myocardial infarction. The pathophysiology of myocarditis in COVID-19 infection is thought to be related to direct viral injury to the cardiac myocytes, but it is also proposed that there may be a component of cytokine storm syndrome (37). Similarly, mechanisms of myocarditis associated with COVID-19 vaccines include but are not limited to molecular mimicry, autoantibody formation, mRNA immune reactivity, trigger of preexisting dysregulated immune processes, and genetic predisposition (38).

Our study have several strengths. First, we studied a large sample size of 58 million individuals. Additionally, various vaccine types were included in this meta-analysis, which allows for generalizability of the relationship between COVID-19 vaccination and myocarditis. Third, due to the high degree of heterogeneity, a random effects meta-analytic framework was invoked.

The findings of this meta-analysis should be interpreted in light of some limitations. First, studies varied in their methods of diagnosing myocarditis: Although myocarditis is suspected by clinical diagnosis, cardiac biomarkers and ECG changes, confirmation is made by performing an endomyocardial biopsy or with a Cardiac MRI (CMR). However, not all medical centers had the facilities to perform CMR or endomyocardial biopsies. Only two studies included three patients who underwent endomyocardial biopsy with no diagnostic evidence of myocarditis on biopsy (4, 17). Another limitation is a wide variation in the follow-up time (range 7–90 days) which might have counfounded the risk estimate. Lastly, although studies from multiple countries were included, most of the patient population were from the US or the UK region. Therefore, the findings may not be generalizable to other geographic regions not studied such as Africa.

## Conclusion

In this systematic review and meta-analysis, we found that the risk of incident myocarditis is more than seven times higher in persons who were infected with the SARS-CoV-2 than in those who received the COVID-19 vaccines. These findings support the continued use of mRNA COVID-19 vaccines among all eligible persons per the CDC and WHO recommendations.

## Data availability statement

The original contributions presented in the study are included in the article/Supplementary material. R code and data to reproduce the results in this article are archived at GitHub. The link to GitHub is: https://github.com/ssentongojeddy/Myocarditis_COVID19. Further inquiries can be directed to the corresponding author.

## Ethics statement

Ethical review and approval was not required for this study in accordance with the local legislation and institutional requirements.

## Author contributions

PS and NV: study concept and design, methodology, supervision, interpretation of data, and drafting of the manuscript. SR: data collection. PS: statistical analysis. All authors critically revised the manuscript for important intellectual content and approved the final version of the manuscript.

## Funding

Penn State College of Medicine Startup Funds to PS. The funder had no role in the design and conduct of the study; collection, management, analysis, and interpretation of the data, preparation, review, or approval of the manuscript, and decision to submit the manuscript for publication.

## Conflict of interest

The authors declare that the research was conducted in the absence of any commercial or financial relationships that could be construed as a potential conflict of interest.

## Publisher's note

All claims expressed in this article are solely those of the authors and do not necessarily represent those of their affiliated organizations, or those of the publisher, the editors and the reviewers. Any product that may be evaluated in this article, or claim that may be made by its manufacturer, is not guaranteed or endorsed by the publisher.
